# Thermally Modulated Specular Phonon Transport in a High‐Debye‐Temperature Diamond Nanobeam

**DOI:** 10.1002/advs.202523242

**Published:** 2026-03-04

**Authors:** Seohee Jang, Seung‐Woo Jeon, Takuma Shiga, Jeeyoung Shin, Sangwook Han, Woosung Park

**Affiliations:** ^1^ School of Mechanical Engineering Hanyang University Seoul South Korea; ^2^ Center for Quantum Technology Korea Institute of Science and Technology Seoul South Korea; ^3^ Department of Advanced Science and Technology Toyota Technological Institute Nagoya Aichi Japan; ^4^ Department of Mechanical Systems Engineering Sookmyung Women's University Seoul South Korea; ^5^ Institute of Advanced Materials and Systems Sookmyung Women's University Seoul South Korea; ^6^ KU‐KIST Graduate School of Converging Science and Technology Korea University Seoul South Korea; ^7^ Division of Quantum Information KIST school Korea University of Science and Technology Seoul South Korea

**Keywords:** boundary scattering, diamond, nanobeams, phonon transport

## Abstract

While the interplay between phonon and boundary dictates thermal transport at the nanoscale, the spectral manipulation of phonon‐boundary scattering is insufficiently demonstrated yet. Here, we choose a single‐crystal diamond nanobeam, a material with one of the highest Debye temperature materials, to investigate the impact of modulated phonon‐boundary scattering with a temperature knob. The thermal conductivity of nanobeams is measured from room temperature down to ∼140 K, and we find that the value monotonically decreases scaling with *T* ^∼1.07^. Compared to the model prediction based on the Boltzmann transport equation combined with ab initio calculation, we find that the experimental data increasingly deviate from the model prediction with diffuse phonon scattering as the temperature decreases. The deviation indicates the increasing portion of non‐diffuse phonon‐boundary scattering. Our analysis indicates that specular phonon‐boundary scattering is more sensitive to temperature in single‐crystal diamond compared to that of silicon. This work suggests that the diamond is a potential material platform to manipulate wave‐like phonon conduction above 100 K.

## Graphical abstract

Thermal transport in single‐crystal diamond nanobeams is measured from 140 to 300 K using electro‐thermal micro‐suspended structures. The thermal conductivity shows an increasing deviation from Boltzmann transport predictions assuming fully diffuse boundary scattering at lower temperatures. This behavior reveals a thermally enhanced contribution of specular phonon–boundary scattering in a high‐Debye‐temperature diamond nanostructure.

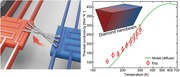

## 1 Introduction

The interplay between thermal phonons and boundaries dictates heat conduction at the nanoscale, which is important in various applications such as light emitting diode [1], extremely integrated circuits [2, 3], and ultra‐high thermal conductivity semiconductors [4, 5, 6, 7, 8]. The surface reflection process of the thermal phonons is generally grouped into either diffuse or specular scattering, and the chance of specular scattering is modeled as a spectral probability [9, 10, 11]. The modulation of specular phonon scattering is a key for coherent manipulation of thermal phonons, and temperature is a potential knob to control the probability [12, 13, 14]. However, the contribution of the specular scattering to thermal transport at temperatures above ∼100 K is insufficiently demonstrated as the spectral probability is nearly insensitive to temperature in silicon [15]. For active modulation of thermal phonons, it is essential to investigate the interplay of phonons and boundaries in other materials platforms [16, 17, 18], such as single crystal diamonds, known for the best thermal conductor with the highest Debye temperature material.

In phonon‐mediated materials, the Debye temperature *Θ*_D_ defines a material‐specific energy scale, providing an approximate cutoff to the vibrational spectrum. At a fixed temperature *T*, the ratio *T/Θ*_D_ indicates the extent of thermal excitation. With decreasing temperature ratio *T/Θ*_D_, high‐frequency phonons become progressively depopulated, and a predominant heat‐carrying phonon spectrum remains to be in the long‐wavelength regime. These long‐wavelength phonons are likely to show quasi‐ballistic transport behavior in a single crystal medium [19]. For Si with a Debye temperature of 645 K [20, 21], cryogenic experiments have been a key method to interrogate quasi‐ballistic phonon characteristics as high‐frequency phonons are frozen [22]. At cryogenic temperature, a primary feature is that phonon‐boundary interactions become more prominent in nanoscale media, leading to boundary‐scattering dominant phonon transport. Much of previous research based on Si has discussed strongly suppressed thermal conductivity in thin films [12, 23], nanowires [24, 25], and nanobeams [26], contributing to building a microscopic model for phonon conduction. The interaction of phonons with boundaries is further discussed in various nanoscopic geometries, such as fishbone‐shaped silicon structures, silicon nanoladders, and nanostructures with kinks, suggesting potential guidelines to tailor phonon conduction via phonon‐boundary interactions. Another feature of cryogenic temperature measurements is to pronounce spectral phonon characteristics as its spectral distribution varies with temperature, where the wave‐like nature and coherence of thermal phonons become increasingly important [27]. Ravichandran et al. investigate the specularity of phonon‐boundary scattering in Si, and the specular probability is experimentally found to be a function of wavelength, following wave‐characteristics [13]. More broadly, recent studies have highlighted that coherent and wavle‐like phonon transport in low‐dimensional and nanostructured systems is typically observable at cryogenic temperatures or in engineered periodic structures [28]. Luckyanova et al. demonstrate coherent phonon transport in GaAs/AlAs superlattices by engineering interface periodicity to preserve phonon phase coherence [17]. Cheaito et al. report a transition from quasi‐ballistic to diffusive transport with increasing temperature from 100 to 300 K by manipulating both the period and total thickness of the AlAs/GaAs superlattice [29]. Chang et al. demonstrate the thermal rectification effect in both carbon nanotubes and boron nitride nanotubes at room temperature by manipulating the nonlinearity of thermal wave transport [30]. Also, wave‐based transport enables directional control of phonon propagation, which can be used for phonon diodes and metagratings [31]. The Abovementioned temperature dependence in spectral distribution of phonons is pronounced only at cryogenic temperatures in silicon, of which the Debye temperature is 645 K. To push forward the limit of spectral modulation of phonons at higher temperatures for practical applications, it is essential to investigate phonon transport in a high Debye temperature material, such as diamond with a Debye temperature of 2230 K [15].

In this work, we investigate the phonon transport of single‐crystal diamond nanobeams of a triangular cross‐section with ∼185 nm in width and ∼120 nm in height. Specifically, we intentionally vary the length of diamond nanobeams from ∼8.4 to ∼34.5 µm to rule out the impact of thermal contact resistance between the samples and their substrate. The measurement temperature ranges from ∼140 to 300 K to capture temperature‐dependent phonon‐boundary scattering characteristics. A Boltzmann transport model combined with ab initio calculations predicts the thermal conductivity of the diamond nanobeams. A combination of the Boltzmann transport model and experimental data unveils the spectral characteristics of phonon transport.

## 2 Sample Preparation

**FIGURE 1:**
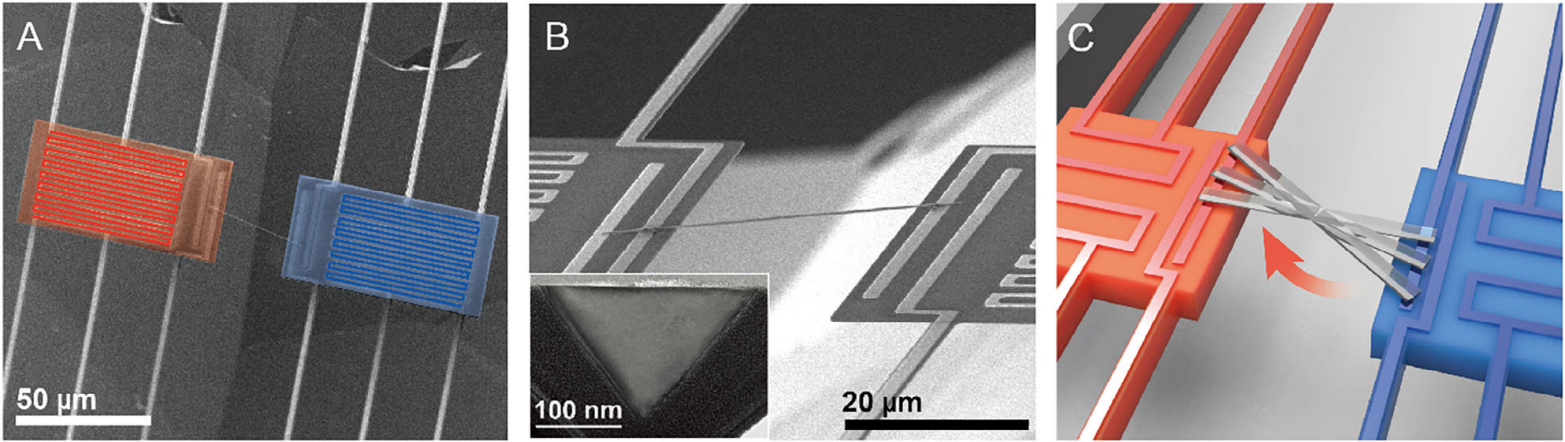
Thermal characterization of diamond nanobeams. (A) Overview of both hot and cold islands for thermal characterization depicted by red and blue colored heater/thermometers, respectively. (B) Zoomed‐in view of the diamond nanobeam sample. Bottom left inset represents a diamond nanobeam with a triangular cross‐sectional area. (C) Schematic of the methodology of varying the length of the diamond nanobeams, not in scale.

The diamond nanobeams are fabricated from a high‐purity single‐crystal diamond along the [100] direction. The nanobeams are placed between two suspended island structures using a nano‐manipulator as seen in Figure 1A. The diamond nanobeams have an isosceles triangular cross‐section with ∼185 nm in width and ∼120 nm in height as seen in the inset of Figure 1B. To remove the impact of thermal contact resistance between the nanobeams and the islands, the sample length varies, ranging from ∼8.4 to ∼34.5 µm, and are placed with a rotation with respect to the suspended islands as illustrated in Figure 1C. To obtain sufficient length dependence, seven diamond nanobeams are measured with the same cross‐sectional area (See Supporting Information for lengths of diamond nanobeam samples). Note that the diamonds have a low defect density of both boron and nitrogen, less than 1 ppb (See Supporting Information for diamond nanobeam fabrication and Transmitted Electron Microscopic image for its cross‐section).

## 3 Thermal Characterization

The diamond nanobeams are characterized using an electro‐thermal characterization method, with suspended two‐island structures. While details of thermal characterization are well‐documented elsewhere [26, 32, 33], briefly, heat is generated using a serpentine heater on an island, and the resulting temperatures of both islands are measured using resistive thermometry. The measured thermal resistance of the sample includes both a volumetric thermal resistance of the nanobeams and the contact resistance between the sample and an electrode [34, 35]. To eliminate the impact of contact resistance, we measure various lengths of the diamond nanobeams with a constant cross‐section. The thermal conductivity *k* is determined from the slope of the measured thermal resistance versus the sample length, and the slope is equal to 1/*kA*, where *A* is the cross‐section. By dividing the cross‐sectional area with the inverse of the slope, we quantify the thermal conductivity of the nanobeam (See Supporting Information for the details of electro‐thermal characterization).

## 4 Results

### 4.1 Temperature Dependence in the Thermal Conductivity of Diamond Nanobeams

**FIGURE 2:**
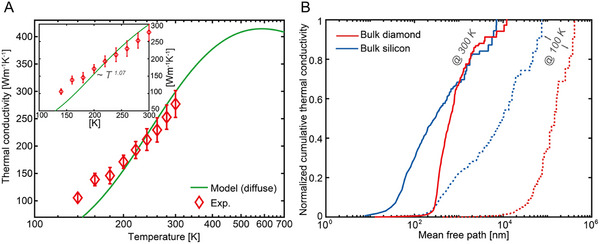
Experimentally measured thermal conductivity and mean free path spectrum. (A) Temperature dependent thermal conductivity for diamond nanobeams from 100 to 300 K. The top left inset depicts experimental values with a first‐principles model within a narrow temperature range, 100 ∼ 300 K. Red hollow diamond markers indicate experimental values of diamond nanobeams with uncertainty, and blue solid line indicates model prediction with the Boltzmann transport model in combination with first‐principles assuming diffuse scattering. (B) Normalized cumulative thermal conductivity as a function of phonon mean free paths for bulk diamond (red) and bulk silicon (blue) at temperatures of 300 K (solid lines) and 100 K (dashed lines), respectively. Each value is normalized by maximum thermal conductivities, which are ∼ 1 x 10^4^ W m^−1^ K^−1^ and ∼ 2 x 10^3^ W m^−1^ K^−1^ for bulk diamond at 100 and 300 K, respectively, and ∼ 685 W m^−1^ K^−1^ and ∼ 123 W m^−1^ K^−1^ for bulk silicon at 100 and 300 K, respectively.

We find that the thermal conductivity of the diamond nanobeams at room temperature is measured to be 277 ± 24 W m^−1^ K^−1^ (See Supporting Information for the details of uncertainty calculations), which is a much suppressed value ∼1/10 of its bulk value [36]. With decreasing temperature, the measured thermal conductivity shows a linear decreasing trend as seen in Figure 2A. Comparing the thermal conductivity with ∼97% of hydraulic cross‐sectional area in Si, the silicon nanowires of circular cross‐sectional area with a diameter of ∼115 nm show a linear decreasing thermal conductivity with decreasing temperature in a much lower temperature region, below ∼100 K [24, 37, 38]. Note that the hydraulic diameter of the diamond with an isosceles triangle shape in this work is ∼115 nm. This linearity indicates that the mean free path of bulk diamond is much longer than that of silicon, as the boundary scattering dictates the mean free path, resulting in the nearly linear temperature dependence of thermal conductivity [39].

### 4.2 Modeling Approaches

To understand the interplay between phonon and boundary, we model the phonon transport using the Boltzmann transport equation (BTE) combined with ab initio calculations [40, 41] (See Supporting Information for the details of phonon transport analysis using first principles). The thermal conductivity of the nanostructure is given by

(1) $$k=\frac{1}{3}\;\int C_{v}\left(\omega \right)v\left(\omega \right)\Lambda_{\mathrm{nano}}\left(\omega \right)\;d\omega$$

(2) $$C_{v}\;\left(\omega \right)=k_{B}\;T\int \frac{\hbar \omega }{k_{B}T}g\left(\omega \right)\frac{\partial f_{B-E}}{\partial T}d\omega$$

where *C*_v_(*ω*) is the spectral specific heat, *v*(*ω*) is the group velocity, *f_B‐E_* (*ω*) is the Bose‐Einstein distribution, *g*(*ω)* is the phonon density of states, *ω* is a phonon angular frequency, and *Λ* is the phonon mean free path (MFP), and the subscript nano denotes the nanobeams, respectively [42]. To obtain the phonon MFP, *Λ*_nano_ *_,_* we use Matthiessen's rule, and the MFP is calculated using

(3) $$\Lambda_{\mathrm{nano}}^{-1}\;\left(\omega \right)=\Lambda_{\mathrm{internal}}^{-1}\;\left(\omega \right)+\Lambda_{\mathrm{boundary}}^{-1}$$

where the subscripts internal and boundary represent internal phonon scattering and phonon‐boundary scattering, respectively [43]. We obtain *Λ*_internal_ from first‐principles calculations as well as *Λ*_boundary_ from the Monte–Carlo simulation [44]. The heat capacity *C*_v_(*ω*) and group velocity *υ*(*ω*) are obtained from ab initio calculations. Given the geometry of our samples, with fully diffuse boundary scattering, *Λ*_boundary_ is estimated to be ∼91 nm (See Supporting Information for the details of Monte–Carlo calculations). The prediction with diffuse phonon boundary scattering agrees with the experimental data within ∼9% at room temperature.

We investigate the MFP spectra of bulk diamond from ab initio calculations by comparing silicon and diamond, as shown in Figure 2B. At 300 K, the heat‐carrying phonons span a narrower range of MFPs while those in silicon show a broad spectrum [45, 46]. Such a concentrated MFP spectrum in diamond over 100 nm range contributes to a much suppressed thermal conductivity compared to that of silicon [47]. At 100 K, diamond shows a still narrower range of MFP spectrum at much longer length compared to that in silicon. This indicates that the suppression in thermal conductivity in nanostructures is likely to be stronger with decreasing temperature [48].

With decreasing temperature, we find that the model prediction increasingly deviates from our experimental values, while the BTE model for silicon nanowires agrees with the experimental data down to cryogenic temperature [49]. We note that the deviation between the experimental data and the model prediction in the temperature range from ∼250 to 300 K is potentially due to both the bulk properties obtained from ab initio calculations as well as the use of Matthiessen's rule for boundary scattering. To capture the increasing deviation between the model prediction and the experimental values with decreasing temperature, we take into account of specular scattering using the Ziman model [9, 50]. Specifically, a specular scattering probability dependent on the phonon wavelength is

(4) $$p_{\lambda }=\;\exp\left(-\frac{16\pi^{2}\eta^{2}}{\lambda^{2}}\right)$$

where *η* is the root‐mean‐square roughness amplitude of the surface, and *λ* is the wavelength of the phonon. To estimate the impact of specularity on the boundary scattering, in Monte‐Carlo calculations, we compare the probability of specular scattering with a random number. If the random number is smaller than the probability of specular scattering, we consider the phonon particle to continue traveling without diffuse scattering. We calculate *Λ*_nano_ as a function of rms surface roughness *η* and phonon wavelength *λ* obtained by Monte‐Carlo simulations.

**FIGURE 3:**
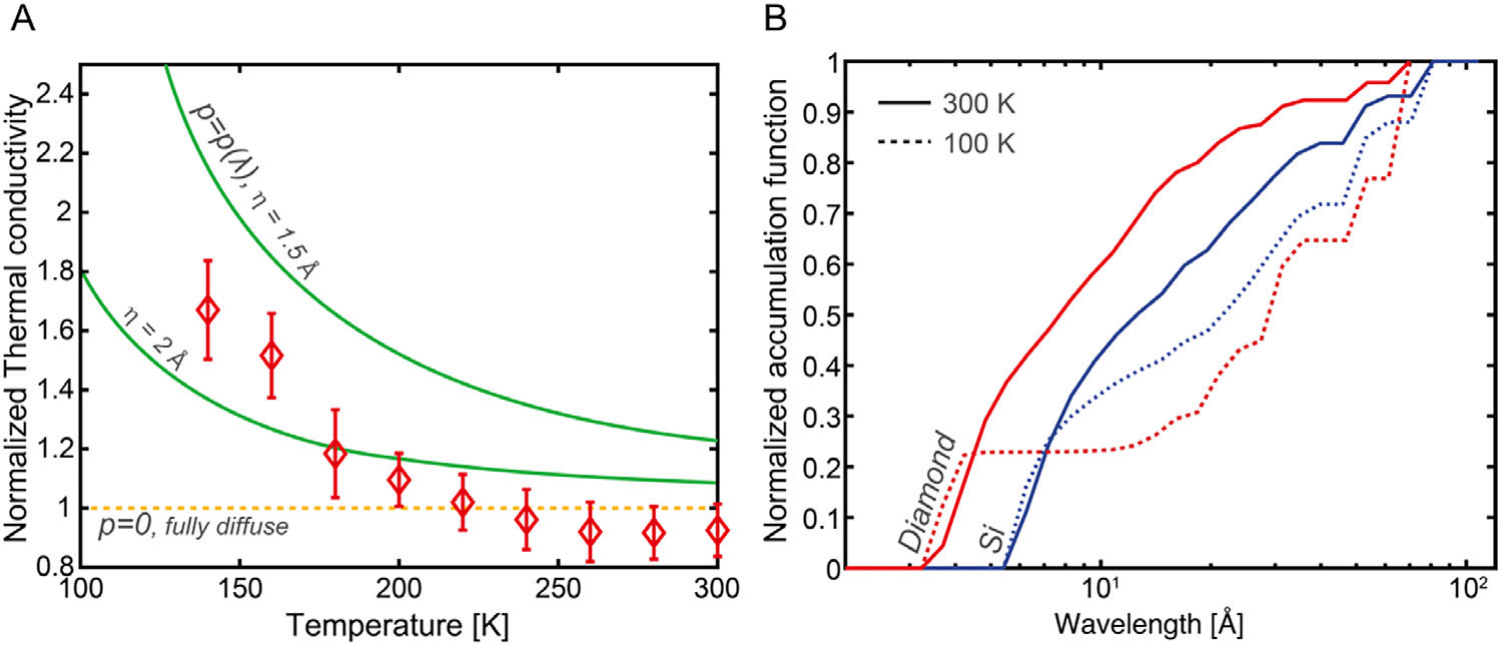
Thermally triggered non‐diffuse scattering and spectral contribution to heat conduction. (A) Normalized thermal conductivity over a temperature range, 100 ∼ 300 K. Each value is normalized by the fully diffuse model prediction. (Color online) Red diamond markers indicate experimental values, green solid lines indicate specular model prediction using the Ziman method with various root‐mean‐square (rms) roughness amplitude of the surface, *η*, from 1.5 to 2 Å, and the yellow dashed line indicates fully diffuse model prediction when the boundary scattering mean free path (MFP) *Λ*_boundary_ = 91 nm. (B) Normalized accumulation functions as a function of phonon wavelength for diamond (red) and silicon (blue) at temperatures of 300 K (solid lines) and 100 K (dashed lines), respectively.

We find that there is an increasing contribution of specular scattering to the thermal conductivity with decreasing temperature. To highlight the specular component, we normalize the experimentally measured thermal conductivity by the model prediction with fully diffuse scattering, as seen in Figure 3A. With decreasing temperature, the measured thermal conductivity is up to ∼40% larger than the model prediction with fully diffuse scattering, indicating a significant portion of specular scattering. The extracted profile of the specular scattering probabilities represents that our nanobeams have root‐mean‐square surface roughness in the range from 1.5 to 2 Å. The experimentally determined roughness is ∼0.95 ± 1.03 Å from transmission electron micrographs, and the value agrees with the roughness from the model prediction (See Supporting Information for surface roughness analysis). For silicon nanofilms, the rms surface roughness value is estimated to be 2.5 ± 0.5 Å [13], suggesting a comparable range of roughness in our diamond nanobeams.

To understand the source of temperature‐dependent specular scattering, we compare the spectral contribution to thermal conductivity in bulk diamond and silicon at 300 and 100 K, as seen in Figure 3B. The comparison suggests that diamond shows stronger temperature dependence in the spectral thermal conductivity distribution compared to that of silicon. At 300 K, we found that the contribution of wavelength is similar for both diamond and Si. At 100 K, however, the striking difference is that most of the heat is carried by phonons in a narrow range of wavelengths in diamond, while Si has a relatively larger span than that of the diamond. The tendency with increasing sensitivity for short‐wavelength phonons has a significant association with the partially specular scattering at the boundary. Hence, the specular reflection of phonons is more prominent in diamond in the low temperature regime, contributing to thermal transport more significantly compared to the counterpart of silicon. Such a strong temperature dependence of thermal transport on wavelength has potential implications for wave‐like phonon transport in nanostructures.

## 5 Conclusion

In this work, we present an experimental study of thermal transport in single‐crystal diamond nanobeams at different temperatures. As an experimental approach, we suggest a novel method to rotate a nanobeam to accommodate varying lengths of the beams, which enables us to remove the impact of contact resistance. This helps us to focus on the thermal transport phenomena in diamond nanobeams. We obtain the temperature‐dependent thermal conductivity, which is suppressed by an order of magnitude relative to bulk values. By combining the Boltzmann transport equation, we examine a close match between experimental and modeled thermal conductivity of diamond nanobeams. It is interesting to observe, however, that below 200 K, we suggest a significant contribution of specular reflection at phonon boundaries in diamond. Moreover, we note that the thermal conductivity of diamond nanobeams shows a transition from the diffuse to specular scattering regime in relatively higher temperature compared to that of silicon nanowires with similar diameters. We provide insights into the boundary scattering characteristics in diamond nanobeams over a range of temperatures that are useful for interpreting thermal measurements on novel diamond 1D nanostructures. Our work opens a new venue to investigate wave‐like phonon transport as well as its active modulation.

## Author Contributions

S.J. and S.W.J. contributed equally to this work. W.P. was responsible for conceptualization. The methodology was carried out by S.W.J., while the investigation was performed by S.J. and T.S. Resources were provided by SH and JS. Supervision was conducted by W.P. The original draft was written by all authors, and all authors also contributed to the review and editing of the manuscript. W.P. conceived the idea and supervised this study. S.J. fabricated the samples. S.J. performed experiments and analyzed the experimental data. T.S. built the statistical model. S.H. provided a discussion for sample fabrications. All authors contributed to writing and editing the article.

## Funding

This research was supported by the National Research Foundation (NRF) funded by the Korean government (MSIT) (Nos. RS‐2024‐00351192 and RS‐2024‐00423772). Part of this research at the National Institute of Advanced Science and Technology (AIST) was financially supported by the JST FOREST (JPMJFR222G) and JSPS KAKENHI (JP23K26055). Part of this research was conducted at the Korea Institute of Science and Technology.

## Conflicts of Interest

The authors declare no conflicts of interest.

## Supporting information




**Supporting File**: advs74489‐sup‐0001‐SuppMat.docx

## Data Availability

All data are available in the main text or the supplementary materials.
