# Supplementary material for: Thermally Modulated Specular Phonon Transport in a High‐Debye‐Temperature Diamond Nanobeam
Source: Adv Sci (Weinh). 2026 Mar 4;13(25):e23242. doi: 10.1002/advs.202523242 (PMC13137799; doi:10.1002/advs.202523242)
Supplement: Supplementary file 1 — Supporting File: advs74489‐sup‐0001‐SuppMat.docx [file ADVS-13-e23242-s001.docx]

**Thermally Modulated Specular Phonon Transport In a High-Debye-Temperature Diamond Nanobeam**

Seohee Jang, Seung-Woo Jeon, Takuma Shiga, Jeeyoung Shin, Sangwook Han and Woosung Park^*^

Seohee Jang, Woosung Park

School of Mechanical Engineering, Hanyang University, Seoul, 04763, South Korea

E-mail: woosungpark@hanyang.ac.kr

Seung-Woo Jeon
Center for Quantum Technology, Korea Institute of Science and Technology, Seoul, 02456, South Korea

Takuma Shiga

Department of Advanced Science and Technology, Toyota Technological Institute, Nagoya, Aichi 468-8511, Japan

Jeeyoung Shin

Department of Mechanical Systems Engineering, Sookmyung Women’s University, Seoul, 04310, South Korea

Institute of Advanced Materials and Systems, Sookmyung Women's University, Seoul, 04310, South Korea

Sangwook Han

Center for Quantum Technology, Korea Institute of Science and Technology, Seoul, 02456, South Korea

KU-KIST Graduate School of Converging Science and Technology, Korea University, Seoul, 02841, South Korea

Division of Quantum Information, KIST school, Korea University of Science and Technology, Seoul, 02792, South Korea

Supporting Information

**Thermally Modulated Specular Phonon Transport In a High-Debye-Temperature Diamond Nanobeam**

Seohee Jang, Seung-Woo Jeon, Takuma Shiga, Jeeyoung Shin, Sangwook Han and Woosung Park^*^

**Supporting Text 1|**

Diamond nanobeam fabrication process

A 350 nm thick SiN_x_ layer is deposited using a high-density plasma enhanced chemical vapor deposition. A 1-dimensional diamond beams are patterned using a negative resist, AR-N 7520.18, Allresist GmbH, by an electron beam lithography. After the e-beam lithography, the pattern is developed using AR 300-47, a TMAH based developer. A dry-etching process is followed to make the SiN_x_ hard mask using the process with SF6 in an Oxford Instruments RIE-100 ICP etcher. Subsequently, a two-step etching processes is applied to release the diamond nanobeam. First, the nanobeams are etched in a normal to the diamond substrate, and etching parameters are 700 W ICP power, 100 W bias power, 45 sccm O_2_ flow rate, 5 sccm Cl_2_ flow rate, and 10 mTorr chamber pressure. Afterward, to undercut the nanobeam, we conduct the etching process using the Faraday cage with a same etching condition except 50 W BIAS power. Additionally, the SiN_x_ hard mask is removed using HF solution. Finally, the nanobeams are annealed at 1100 ˚C for 2 hours under high vacuum to recover surface roughness. The overall process is illustrated in **Figure S1**.

**
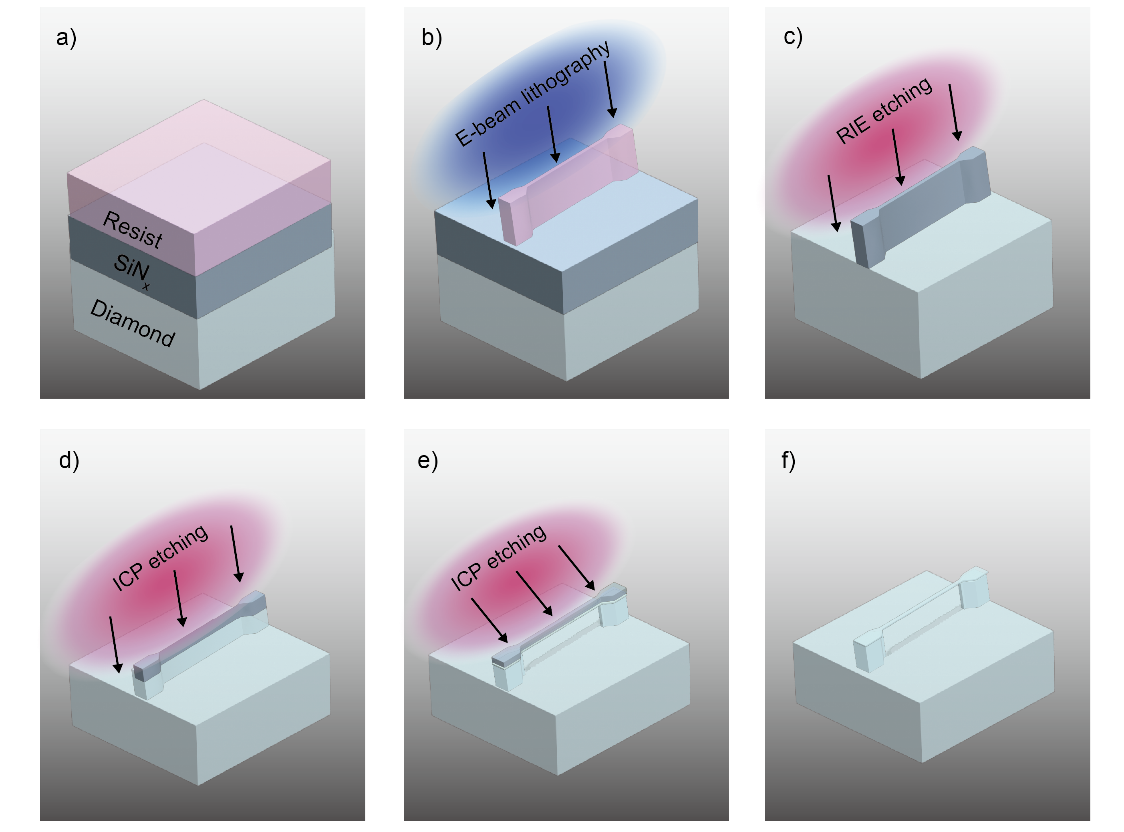
**

**Figure S1.**

Illustrated fabrication process of diamond nanobeams. a) Silicon nitride deposition on a bare diamond substrate, followed by spin coating of a negative e-beam resist. b) A hard mask pattern using electron beam lithography. c) Reactive ion etching for silicon nitride layer. d) Vertical etching of diamond. e) Angled etching of diamond to undercut. f) HF cleaning to remove the nitride layer.

**
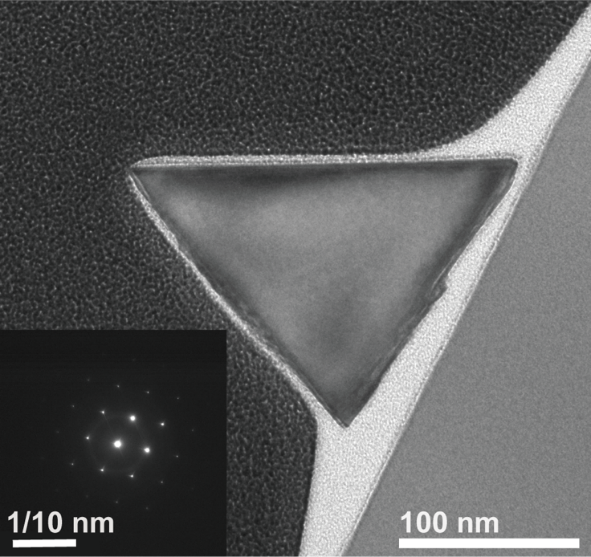
**

**Figure S2.**

A transmission electron micrograph for the cross-section of a diamond nanobeam. The inset shows a fast Fourier transform pattern, indicating a single crystal.


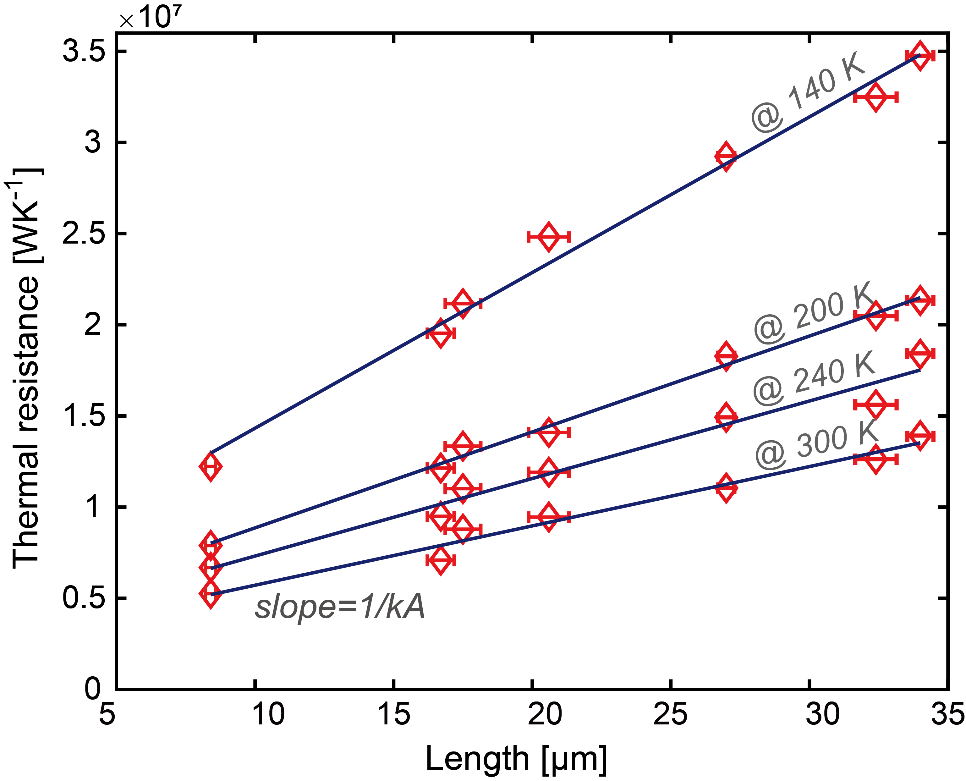


**Figure S3.**

Experimentally measured thermal resistance for diamond nanobeams with varying lengths, ranging from 8.4 μm to 34.5 μm. Red diamond markers are experimental values with uncertainty of each length. Green solid lines are obtained by linear fitting of experimental values, and the slope is 1/*kA*, where A is a cross sectional area of the nanobeams.


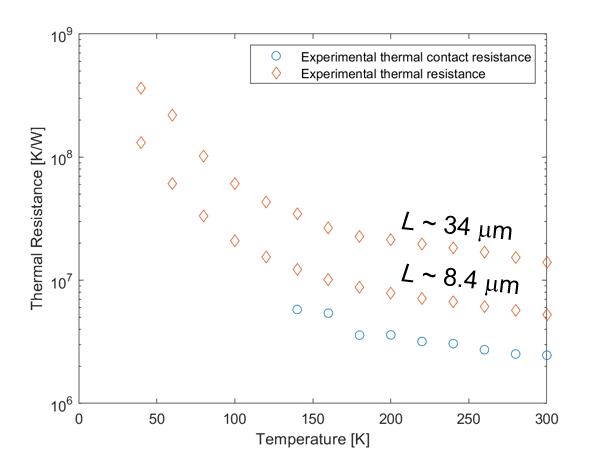


**Figure S4.**

Temperature dependence of the experimentally measured thermal resistance for diamond nanobeams with two different beam lengths, *L* ~ 8.4 μm and *L* ~ 34.5 μm. Note that the thermal resistance includes the thermal contact resistance. Also, the extracted thermal contact resistance from the beams with various lengths is added in blue hollow circle. The thermal contact resistance increases rapidly with decreasing temperature.**Supporting Text 2|**

Error bars

We quantify the uncertainty of our measurements using

|  | $\left( \frac{\Delta k}{k} \right)_{Total}=\sqrt{\left( \left( \frac{\Delta k_{var,1}}{k} \right)^{2}+\left( \frac{\Delta k_{var,2}}{k} \right)^{2}+\cdots\right)}$ | (2) |
| --- | --- | --- |

where *k* is the thermal conductivity, and $var$ indicates each component contributing to the uncertainty. The uncertainty is primarily due to the uncertainty of the beam geometry. Each contribution is summarized in **Table S1**. The total error is less than ~10.2 % of the thermal conductivity, corresponding to 277 ± 16 W⋅m^-1^⋅K^-1^ at room temperature.

**Table S1.**

Measurement uncertainties in this work.

|  | Cross-sectional area  (m^2^) | Beam length  (μm) | The propagated uncertainty in thermal conductivity (Wm^-1^K^-1^) |
| --- | --- | --- | --- |
| Uncertainty  (%) | 6.5 | 2.1 – 7.8 | 7.0 – 10.2 |

**Table S2.**

Measurement uncertainty of the length scale.

| *L* (μm) | ~8.8 | ~17.8 | ~18.3 | ~21.1 | ~28.1 | ~30.7 | ~34.5 |
| --- | --- | --- | --- | --- | --- | --- | --- |
| Uncertainty (%) | ~4.3 | ~5.6 | ~7.8 | ~7.1 | ~2.1 | ~5.0 | ~2.8 |

**Supporting Text 3|**

*Ab initio* calculation for a bulk medium

Phonon transport properties in bulk crystalline diamond and silicon are evaluated by solving the linearized Boltzmann transport equation using interatomic force constants (IFCs) derived from first-principles calculations. After structural optimization of the primitive unit cell of each material, harmonic IFCs are obtained via density functional perturbation theory (DFPT) on the optimized cell with a 10ⅹ10ⅹ10 q-point mesh. For the anharmonic part, third-order IFCs are calculated using the finite-displacement method on a 4ⅹ4ⅹ4 supercell built from the primitive unit cell. The cutoff for third-order interactions is set following pervious literature,^[1, 2]^ including up to the 3^rd^ nearest neighbors for silicon and up to 10^th^ nearest neighbors for diamond. The conditions used in the DFT/DFPT calculations, such as pseudopotentials and cutoff energy of plane-wave, are similar to those in the aforementioned references. Intrinsic phonon scattering mechanisms is considered including three-phonon and phonon-isotope scatterings with natural isotope concentrations.^[3]^ For diamond, a full iterative solution of the BTE is used. All calculations are carried out using the Quantum ESPRESSO and ShengBTE packages.^[4, 5]^

Monte-Carlo simulation

A computational domain is defined as seen in in **Figure S5**

, and the boundaries of a nanobeam is defined using a normal vector ***n*** and two tangential vectors ***t***_1_, ***t***_2_. The relevant vectors are summaries in **Table S3**. 5000 number of phonons are generated at a random location *P*_0_ *(P_0, x_, P_0, y_, P_0, z_)* on a x-z plane, noted as the plane 4. The initial location *P*_0_ is defined using three vertices *A*, *B*, *C* and two random numbers *r*_1_, *r*_2_

|  | $A=\left( \frac{W}{2}, 0, 0 \right), B=\left( -\frac{W}{2},0, 0 \right), C=\left( 0, 0, H \right) ,$ | (5) |
| --- | --- | --- |
|  | $P_{0}=(1-\sqrt{r_{1}})A+\left( 1-r_{2} \right)\sqrt{r_{1}}B+r_{2}\sqrt{r_{1}}C.$ | (6) |

where *W* and *H* denote the width and height of the triangle, respectively.^[6]^ The directions of the phonons are assigned random direction ***D***(*D*_x_*, D*_y_*, D*_z_) with angular uniformity using two random numbers *r*_3_, *r*_4_

|  | $D_{x}=\cos\theta\boldsymbol{n}$ | (7) |
| --- | --- | --- |
|  | $D_{y}=\sin\theta\cdot\cos\phi\boldsymbol{t}_{1}$ |  |
|  | $D_{z}=\sin\theta\cdot\sin\phi\boldsymbol{t}_{\boldsymbol{2}}\boldsymbol{,}$ |  |

where sin*θ* = *r*_3_, *φ* = 2π*r*_4_.^[7]^ The phonons travel until hit the boundaries, and the new location *P*_1_*(P*_1 ,x_*, P*_1, y_*, P*_1, z_*)* is updated using the intersection.

To calculate the direction after scattering, a random number is drawn to determine whether phonons scatter diffusely or specularly. If a random number is smaller than a specularlity *p*, the direction is defined as

|  | $D_{x}=-\cos\theta\boldsymbol{n}$ | (8) |
| --- | --- | --- |
|  | $D_{y}=\sin\theta\cdot\cos\phi\boldsymbol{t}_{1}$ |  |
|  | $D_{z}=\sin\theta\cdot\sin\phi\boldsymbol{t}_{\boldsymbol{2}}\boldsymbol{,}$ |  |

with incident ray angle *θ* and *φ*. The specular scattering is not counted as an effective scattering. The traveling distance between the points is calculated to be *Λ*_ray_

|  | $\Lambda_{ray}=\frac{\left( P_{plane}-P_{0} \right)\cdot\boldsymbol{n}}{\boldsymbol{n}\cdot\boldsymbol{D}}\boldsymbol{,}$ | (9) |
| --- | --- | --- |

where *P*_plane_ is any point in boundary plane. The updated position *P*_1_ is calculated as

|  | $P_{1}= P_{0}+\Lambda_{ray}\cdot\boldsymbol{D}.$ | (10) |
| --- | --- | --- |

after 5000 of phonon simulations, the phonon mean free path *Λ*_boundary_ is obtained by averaging the travel distance. The cumulative distribution function of phonon travel distance between boundaries in **Figure S6**. The distribution of *Λ*_boundary_ obtained by Monte-Carlo simulation results is used as a boundary scattering length.

**Table S3.**

**n** and **t** vectors used for defining boundaries.

|  | Normal vector ***n***(n_x_, n_y_, n_z_) | Tangential vector 1 ***t***_1_(t_1, x_, t_1, y_, t_1, z_) | Tangential vector 2 ***t***_2_(t_2, x_, t_2, y_, t_2, z_) |
| --- | --- | --- | --- |
| Plane 1 | [0, 0, -1] | [0, 1, 0] | [1, 0, 0] |
| Plane 2 | [cos(141.9°), 0, sin(141.9°)] | [0, 1, 0] | [cos(232.1°), 0, sin(232.1°)] |
| Plane 3 | [cos(37.4°), 0, sin(37.4°)] | [0, 1, 0] | [cos(127.4°), 0, sin(127.4°)] |
| Plane 4 | [0, 1, 0] | [1, 0, 0] | [0, 0, 1] |
| Plane 5 | [0, -1, 0] | [1, 0, 0] | [0, 0, 1] |

**
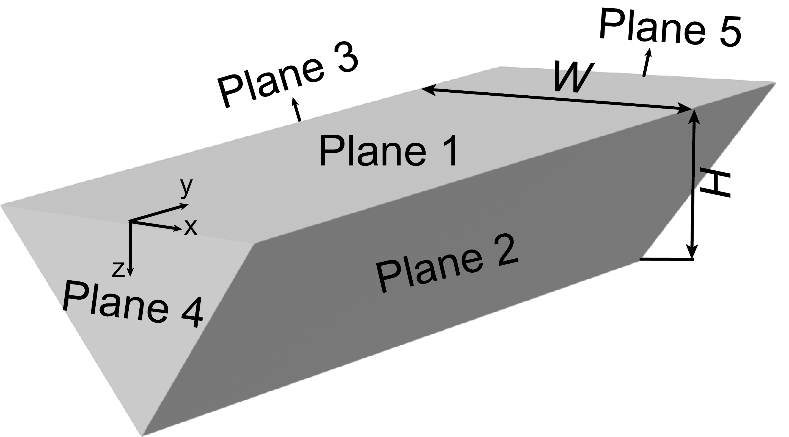
**

**Figure S5.**

Monte-Carlo simulation geometry.


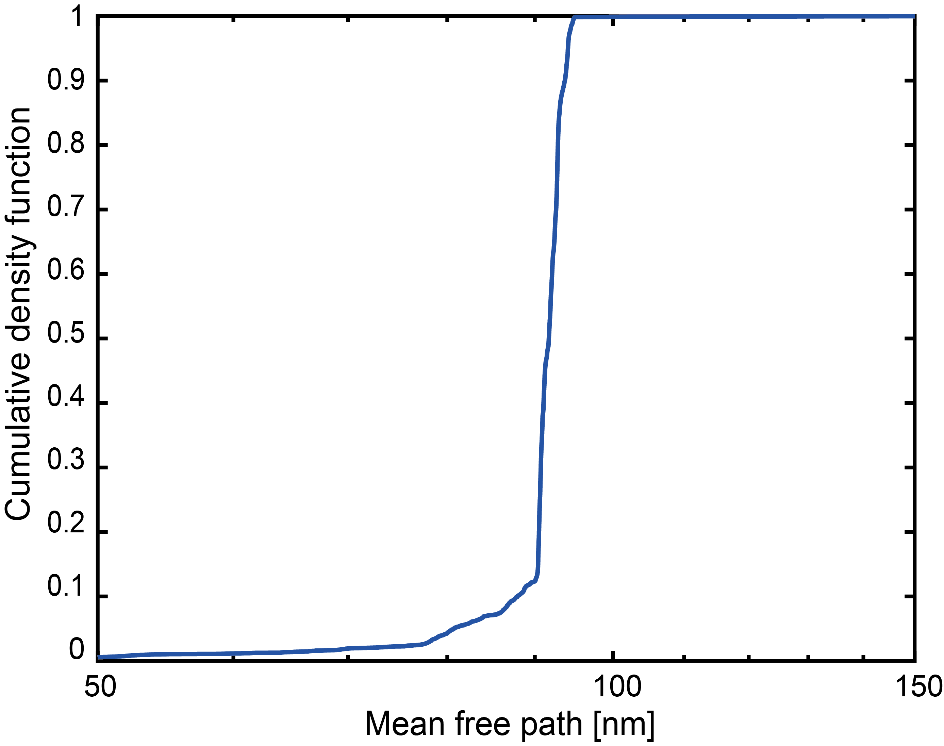


**Figure S6.**

Cumulative density function represents the probability of occurrence of boundary mean free paths calculated by Monte-Carlo simulation with 5000 iterations.

**Supporting Text 4|**

Surface roughness analysis

High-resolution transmission electron micrographs of the nanobeam cross-section are used to quantify the roughness of the diamond sidewall. In the electron micrographs, we define the boundary of the nanobeams using the transition of ist contrast. Specifically, the diamond boundary is defined as the location of the maximum grayscale intensity gradient across the interface as seen in **Figure S7**. With the defined the interface, the root mean square roughness is calculated to be ~0.95 ± 1.03 Å. The uncertainty is noticeable in this roughness calculations as the boundary is not perfectly sharp in the transmission electron micrographs. The grayscale transition near the boundary is broadened by overlapped material information along the electron beam path, leading to a finite-width blurred boundary. This dark locus along the boundary is considered as a source uncertainty, which shows a shade region in **Figure S7 c)**.


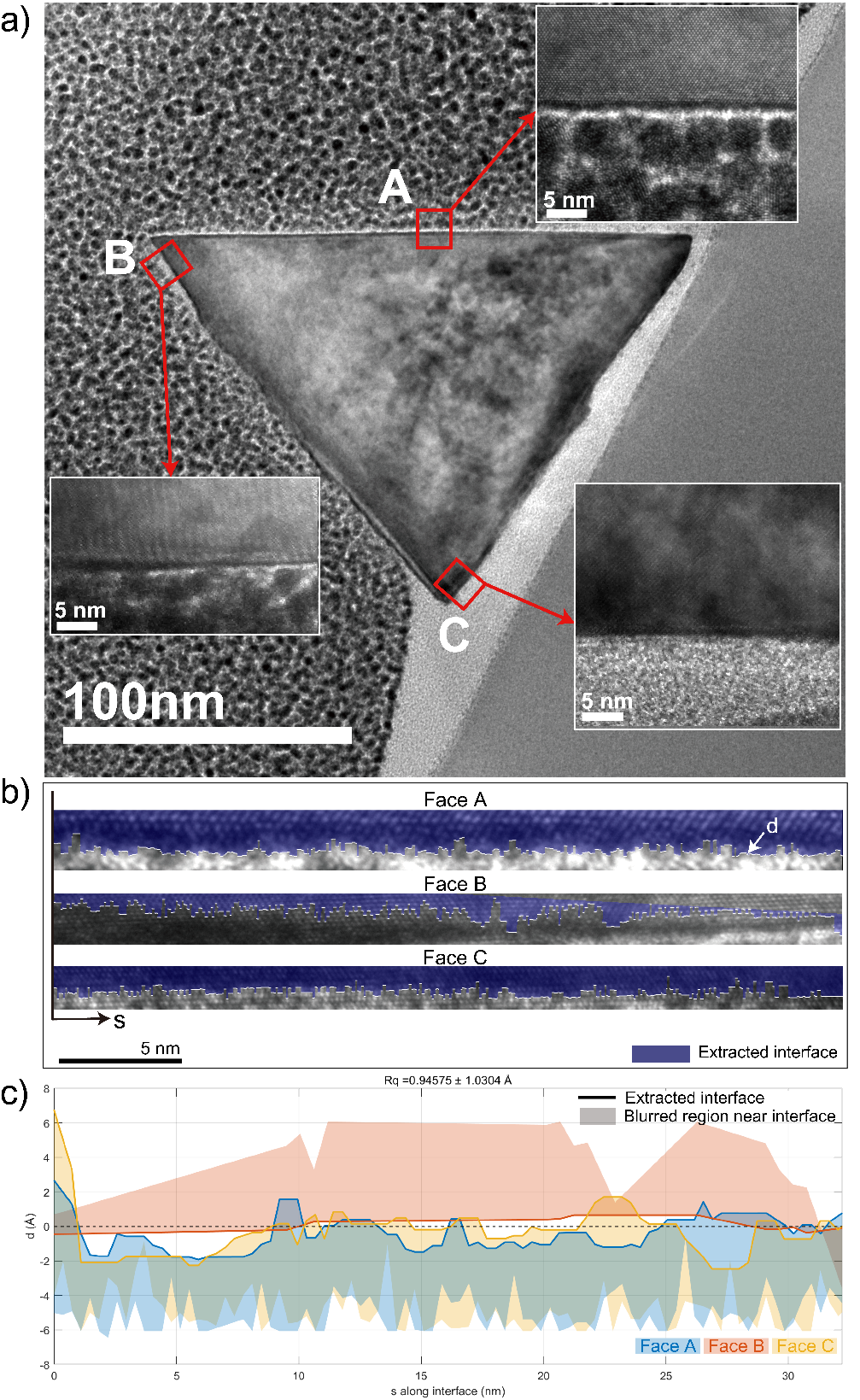


**Figure S7.** a) High-resolution transmission electron micrographs for the cross section of the diamond nanobeam, and specific spots used for roughness quantification are shown in insets as labeld from A to C. b) Extracted diamond boundary traces for facets A–C, where *s* denotes the distance along the mean interface line. c) Normal displacement profiles *d*(*s*) and the shaded blurred region near the interface derived from the darkest-locus method, yielding an overall roughness to be ~0.95 ± 1.03 Å.

**References**

[1] Li, Wu, Natalio Mingo, Lucas Lindsay*, et al.* "Thermal Conductivity of Diamond Nanowires from First Principles." *Physical Review B—Condensed Matter and Materials Physics* 85, no. 19 (2012): 195436.

<https://doi.org/10.1103/PhysRevB.91.134306>.

[2] Lindsay, L., D. A. Broido, and T. L. Reinecke. "Ab Initiothermal Transport in Compound Semiconductors." *Physical Review B* 87, no. 16 (04/02/ 2013): 165201.

<https://doi.org/10.1103/PhysRevB.87.165201>.

[3] Tamura, Shin-ichiro. "Isotope Scattering of Dispersive Phonons in Ge." *Physical Review B* 27, no. 2 (01/15/ 1983): 858-66.

<https://doi.org/10.1103/PhysRevB.27.858>.

[4] Giannozzi, P., O. Andreussi, T. Brumme*, et al.* "Advanced Capabilities for Materials Modelling with Quantum Espresso." *J Phys Condens Matter* 29, no. 46 (Nov 22 2017): 465901.

<https://doi.org/10.1088/1361-648X/aa8f79>.

[5] Li, Wu, Jesús Carrete, Nebil A. Katcho, and Natalio Mingo. "Shengbte: A Solver of the Boltzmann Transport Equation for Phonons." *Computer Physics Communications* 185, no. 6 (2014/06/01/ 2014): 1747-58.

<https://doi.org/10.1016/j.cpc.2014.02.015>.

[6] Osada, Robert, Thomas Funkhouser, Bernard Chazelle, and David Dobkin. "Shape Distributions." *ACM Transactions on Graphics (TOG)* 21, no. 4 (2002): 807-32.

[7] Kukita, K., and Y. Kamakura. "Monte Carlo Simulation of Phonon Transport in Silicon Including a Realistic Dispersion Relation." *Journal of Applied Physics* 114, no. 15 (2013).

<https://doi.org/10.1063/1.4826367>.
